# Spatial and temporal data to study residential heat decarbonisation pathways in England and Wales

**DOI:** 10.1038/s41597-022-01356-9

**Published:** 2022-05-27

**Authors:** Alexandre Canet, Meysam Qadrdan, Nick Jenkins, Jianzhong Wu

**Affiliations:** School of Engineering, Cardiff University, Cardiff, CF24 3AA UK

**Keywords:** Energy modelling, Energy economics, Energy grids and networks, Energy supply and demand

## Abstract

The decarbonisation of residential heating is crucial if the net-zero target in the United Kingdom is going to be achieved. This paper describes methods to produce data to quantify the impacts of residential heat decarbonisation on the energy supply infrastructure across England and Wales. For the year 2018, annual heat demand for a range of dwellings was estimated for almost 35,000 local areas (known as Lower Layer Super Output Areas: LSOAs). Energy savings through implementing the potential energy efficiency measures and the indicative costs of the energy efficiency measures were quantified. Profiles were synthesised for heat production and energy demand of selected heating technologies using average daily temperature and data from trial projects. These profiles were created to study the impacts of different types of heating technology in each LSOA under user-defined heat decarbonisation pathways. Data describing the dwelling stock, heating technologies, annual heat demand for each LSOA, indicative costs of energy efficiency improvements for each local authority and the profiles for each technology were created.

## Abstract (summary)

**Table:** 

Measurement(s)	Annual heat demand • Half-hourly heat production of ASHPs, GSHPs, gas boilers and resistance heaters • Half-hourly energy consumption of ASHPs, GSHPs, gas boilers and resistance heaters • Costs of energy efficiency
Technology Type(s)	digital curation • supervised machine learning
Sample Characteristic - Environment	anthropogenic environment
Sample Characteristic - Location	England • Wales

## Background & Summary

Decarbonising heat is a major challenge facing UK energy policy. In 2018, residential space heating and hot water were responsible for 394 TWh of the final energy consumption of the UK. This is approximately 41% of total UK final energy consumption across all sectors (excluding transport)^1^. This is the equivalent of more than 78 MtCO_2_e (calculated using carbon emissions factors^2^). Currently, residential heating in the UK is mostly reliant on natural gas. In 2018, 302 TWh of natural gas was consumed for space heating and hot water. To achieve its net-zero target by 2050, the UK residential heat sector is expected to undergo a radical change to significantly increase the installation of heat pumps and hydrogen boilers. However, there are two key questions regarding the decarbonisation of the residential heat sector: (1) What are the suitable technological options for heat decarbonisation? and, (2) what are the impacts of various heat decarbonisation pathways on the local and national energy supply infrastructure? See Table 1 for a glossary of terms and acronyms used in this paper.

**Table 1:** Glossary of terms and acronyms.

Term	Definition
ASHP	Air-source heat pump.
EPC	Energy Performance Certificate, which provides details of the energy performance of a property.
COP	Coefficient of performance.
Gas boiler	In this paper, gas boiler refers to a boiler that uses natural gas or hydrogen as a fuel.
GSHP	Ground-source heat pump.
LSOAs	Lower layer Super Output Areas, which give the boundaries of geographical areas that are used to organise national statistics and census data from the Office for National Statistics. An LSOA has on average 1,614 inhabitants and 672 households. There are 34,753 LSOAs across England and Wales^29^.
MSOAs	Middle layer Super Output Areas, which give the boundaries of geographical areas that are used to organise national statistics and census data from the Office for National Statistics. An MSOA is constituted from several LSOAs. There are on average 4.8 LSOAs in each MSOA, for a total of 7,201 MSOAs in England and Wales^29^.
OAT	Outside air temperature.
ONS	The Office for National Statistics is in charge of producing official statistics for the UK.
UK	The United Kingdom includes England, Northern Ireland, Wales and Scotland. Great Britain is the UK excluding Northern Ireland.

### Technological options for low carbon heating

The pathways for heat decarbonisation presented in the literature^3,4^ focus on national-level changes and trends. These studies use national and regional energy models to suggest a mix of heating technologies for a given pathway and to calculate the impacts of these heating technologies on the energy system. The two heat decarbonisation pathways that have been identified are ‘electrification’ and ‘hydrogen’. In the ‘electrification’ pathway, heat demand is met mainly by air-source and ground-source heat pumps, and by resistive heating. In the ‘hydrogen’ pathway, heat demand is primarily met from hydrogen boilers. The national-level heat decarbonisation pathways can provide insights into what the future of heat could look like, and they indicate the scale of the changes that are required to be undertaken by the energy system. These pathways support the creation of national-level strategies, policy actions and relevant incentives for heat decarbonisation. However, the uptake of suitable and practical heating technologies in local areas depends on local circumstances.

### Impacts of heat decarbonisation on energy supply infrastructure

Several studies have been carried out to estimate the future heat demand of the UK. These studies provide many useful insights into the temporal characteristics of aggregate heat demand (e.g., peak and variation of heat demand) for the whole of Great Britain^5^. However, the shape and magnitude of the heat demand profiles could be significantly different from one local area to another, depending on the characteristics of the buildings and the mix of heating technologies. To quantify the impacts of different heat decarbonisation pathways on the energy supply infrastructure, at local as well as national scales, it is crucial to consider spatial and temporal data for heat demand.

In this work, we have created a database^6^ that includes data such as dwelling type, heating system, dwelling energy efficiency, annual heat demand, normalised half-hourly heat production and energy demand of heating technologies for each LSOA in England and Wales.

**Fig. 1:**
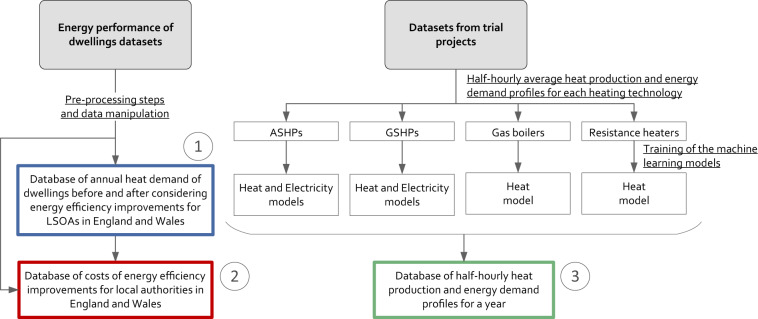
Overview of the methodologies used to produce the datasets of this study.

Figure 1 gives an overview of the methods that have been used to produce the three datasets forming this database. The annual heat demand for different type of dwellings before and after considering energy efficiency improvements (dataset 1) was derived from publicly available information about the energy performance of dwellings. Dataset 2 is an extension of dataset 1 and includes data about the costs of implementing these energy efficiency improvements. Dataset 3 was created using synthesised half-hourly heat production and energy demand of four heating technologies, as follows: air-source heat pumps (ASHPs), ground-source heat pumps (GSHPs), gas boilers (both natural gas and hydrogen boilers) and resistance heaters. These profiles were created using machine learning models that were trained using datasets from trial projects where the heat production and/or energy demand of these four heating technologies were recorded.

## Methods

### Annual heat demand of dwellings

The building stock data and energy performance information of dwellings (obtained from EPCs) were used to estimate the annual heat demand of dwellings in a LSOA. The annual heat demand of the building stock, with and without energy efficiency improvements, was calculated for all of the LSOAs in England and Wales for 16 dwelling categories. In this study, a *dwelling category* is the combination of a dwelling type (i.e., detached, semi-detached, terraced, or flat) and a heating system (i.e., natural gas boiler, resistance heater, biomass boiler or oil boiler).

**Fig. 2:**
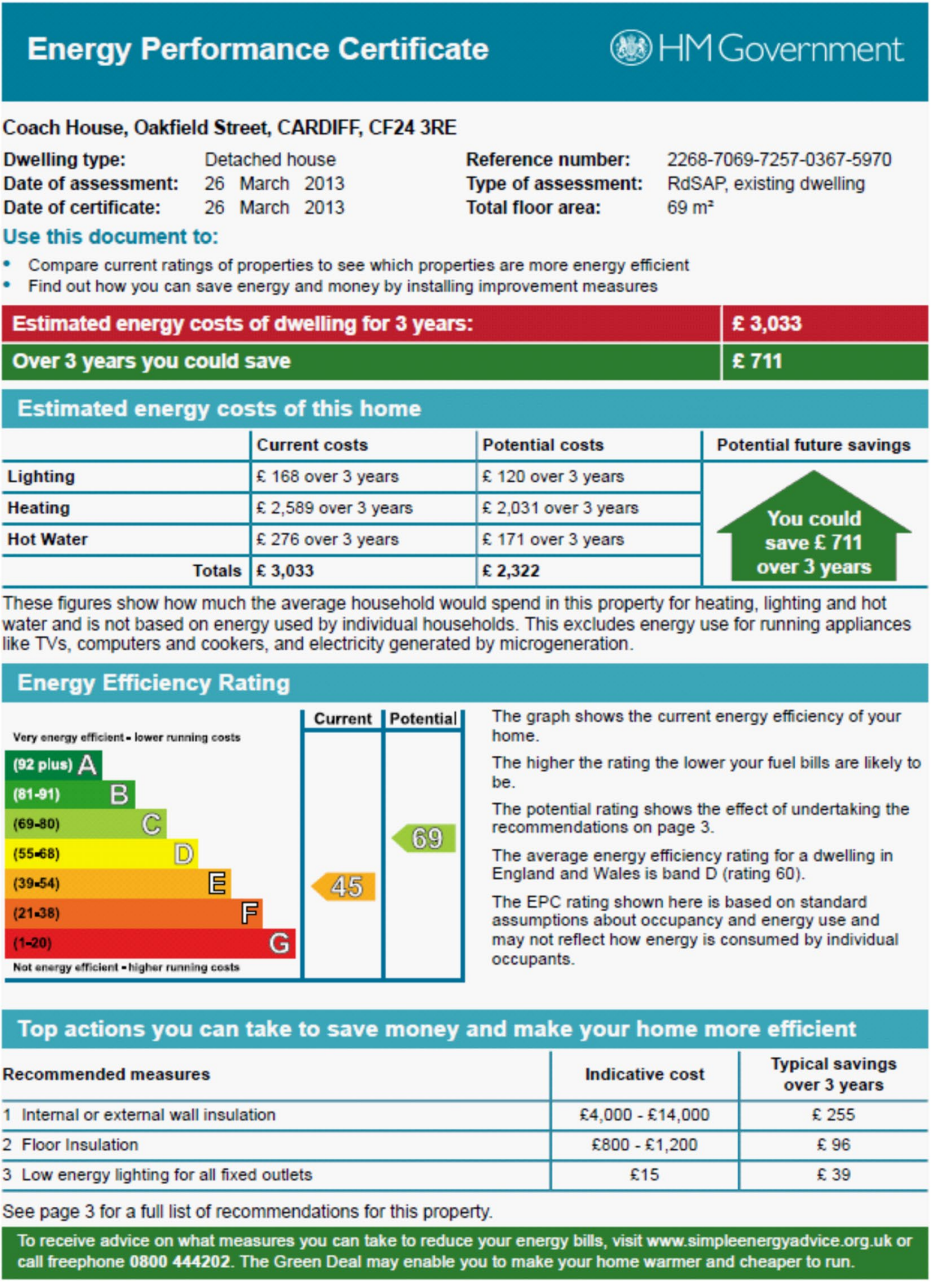
Example of an energy performance certificate for a detached house^7^.

Energy Performance Certificates (EPCs) were used to estimate the annual heat demand of the 16 dwelling categories within a local area for a typical year. Figure 2 shows an EPC for a detached house with a current energy efficiency rating of 45 (band E ∈ [39,54]) and a potential energy efficiency rating of 69 (band C ∈ [69,80]) on a scale from 1 (band G ∈ [1,20]), the worst, up to 100 (band A ∈ [92,100]), the best. The space heating and hot water demand considering current and potential energy efficiency ratings are also estimated in an EPC. The annual heat demand based on potential energy efficiency ratings considers that all of the recommended energy efficiency measures impacting heat demand in the EPC have been implemented.

The EPC database for all local authorities (with spatial resolution at postcode level) in England and Wales was downloaded from the open data communities platform^7^. The data were cleaned, and outliers and duplicates were removed. The cleaning process entailed:

1. Only keeping the most recent EPC for dwellings with multiple EPCs. This was done by filtering the database using the building reference number and the date of the EPC.
2. Removing the EPCs where the potential heating cost is more than 10% higher than the current heating cost. For a dwelling, the energy efficiency measures recommended on an EPC can decrease the heat gains from appliances (i.e., switching to LED lights) increasing the heat demand to be supplied by the heating system and thus the heating cost.
3. Using 15 kWh/m^2^ as the minimum threshold for the heat consumption of a dwelling, which is equivalent to a passivHus requirement.
4. Using 400 kWh/m^2^ as the maximum threshold for the heat consumption in a dwelling.
5. Removing the dwellings with an unspecified number of rooms.
6. Grouping the EPCs into dwelling categories. The dwellings’ postcodes were also used to link them to an LSOA, MSOA and LA using a lookup table published by the ONS^8^.

**Fig. 3:**
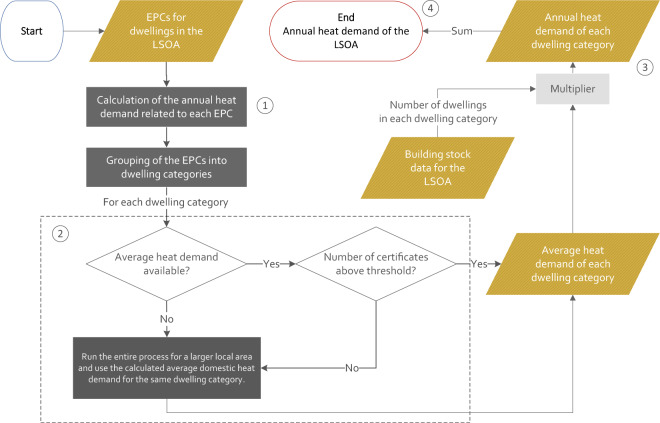
Diagram of the process used to estimate the heat demand of an LSOA.

Figure 3 illustrates the methodology that was used to calculate the residential annual heat demand of a LSOA using the cleaned EPC database, which includes the following steps:

1. The average annual heat demand was calculated for each dwelling category from the information displayed on the EPC.
2. Given that not all the dwellings in a LSOA have an EPC, when the number of EPCs for a dwelling category was too low to estimate the average heat demand, the average heat demand for this dwelling category was calculated by running steps 1 to 3 for an extended geographical area (i.e., MSOA, LA, and Country).
3. The annual heat demand of each dwelling category was calculated.
4. The residential heat demand of a LSOA was calculated by aggregating the annual heat demand of all the dwelling categories in this LSOA.

Hereafter, this will be referred to as the *EPC-based* method in this paper.

### Half-hourly heat production and energy demand of individual heating technologies

XGboost^9^, which is an extreme gradient boosting algorithm, was used to create the machine learning models to synthesise half-hourly heat production and half-hourly energy demand for each heating technology, as follows:

- Because their coefficient of performance (COP) changes with the outside air temperature (OAT), two models were created for ASHPs: the first to synthesise half-hourly heat production and the second to synthesise half-hourly electricity demand.
- Because their COP changes with the temperature of their heat sources which is linked to the OAT, two models were created for GSHPs: the first to synthesise half-hourly heat production, and the second to synthesise half-hourly electricity demand.
- A single model was created for gas boilers, which synthesised half-hourly gas demand. The half-hourly production was derived from the half-hourly gas demand by considering a constant average efficiency of the heating systems of 84%^10^.
- A single model was created for resistance heaters, which synthesised half-hourly electricity demand for heating. The half-hourly production was derived from the half-hourly gas demand by considering a constant efficiency of the heating systems of 100%.

The models were trained using time series data from residential heating trial projects (introduced in Section 0) considering the most influential variables, including time and OAT.

#### Input data to the machine learning models

A dataset of half-hourly aggregated average heat production and energy demand from dwellings for each of the four heating technologies was used to test and train the machine learning models.

#### Collection of the data

Four datasets from trial projects were used in this study. These datasets include records from heat production and electricity demand data of ASHPs, GSHPS and resistance heaters, as well as the gas demand data of gas boilers. Table 2 give the information for the trial datasets the number of dwellings recorded, the geographic area and the duration of the metering.

**Table 2:** Details of the trial datasets for each technology used in this project.

Heating technologies	Sources	Number of dwellings with data recorded*	Geographic area	Duration of the metering	Type of dwellings	Granularity of the original data
ASHPs	RHPP scheme^30^	29 to 251	UK	01/10/2013 to 28/02/2015	Social housing	2 min
GSHPs	RHPP scheme^30^	11 to 78	UK	01/10/2013 to 30/09/2014	Social housing	2 min
Gas boilers	EDRP^31^	>8,000	UK	01/07/2009 to 30/09/2010	A total of 18 types of households representing different UK customers	30 min
Resistance heaters	EDRP^31^	>14,000	UK	01/06/2009 to 31/06/2010	A total of 18 types of households representing different UK customers	30 min

RHPP: Renewable Heat Premium Payment scheme, EDRP: Energy Demand Research Project. *This column gives the number of dwellings for which data were recorded for each time step of the dataset. Due to missing data and different recording periods for each dwelling (e.g., some dwellings joined the trial later than the others), the number of dwellings with data recorded at each time step is not constant.

#### Creation of the half-hourly profiles

For resistance heaters, some preliminary steps were required to calculate the half-hourly electricity demand for heating. The Energy Demand Research Project published electricity demand at half-hourly resolution for 14,000 dwellings from early-2008 to the end of 2010. No information was provided regarding the type of heating system in each household. Hence, to determine if a household was using resistance heaters or not, the average daily electricity demand in summer and winter were compared. If the demand in winter was at least twice that in summer, then the household was considered to be electrically heated. Two separate sub-datasets were created: the first was a dataset for electrically heated households (3,367 dwellings) and the second was a dataset for households that used energy carriers other than electricity for heating (10,952 dwellings). The difference between the average aggregated half-hourly electricity demand of these sub-datasets was used to represent a half-hourly profile of the electricity used by resistance heaters for heating. For the ASHPs, GSHPs and gas boilers, the datasets were directly used to create aggregated average half-hourly heat production and energy demand of these heating technologies. The UK daily average outside air temperature (OAT) data from the BMRS website^11^ was also added to the profiles.

#### Cleaning the half-hourly profiles

In terms of cleaning procedure, the aggregated average profiles for ASHPs and GSHPs were cleaned using a rolling z-score on the heat production data with a window size of 24. Entries with an absolute z-score above 3 were removed from the dataset. Further data was removed manually. For ASHPs, 338 entries were removed from the training dataset. For GSHPs, 146 entries were removed from the training dataset. For gas boilers and resistance heaters, no procedure was performed to clean the data. Table 3 shows the number of entries in the final datasets.

**Table 3:** Number of entries in the datasets for each heating technology.

Heating technologies	Number of entries in the dataset
ASHPs	24,430
GSHPs	17,393
Gas boilers	21,936
Resistance heaters	18,192

Note: The final datasets are based on the aggregation of profiles from individual dwellings; thus, it can be assumed that these profiles embed the after diversity maximum demand (ADMD). This is also the case of the models trained using these datasets in this study and the profiles published.

#### Testing and training the models

**Fig. 4:**
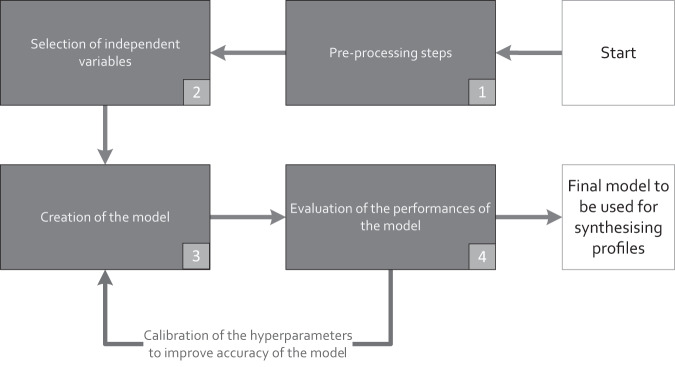
Flow diagram showing the steps to create the machine learning models of this study.

Figure 4 shows an overview of the steps that were followed to create the models.

It includes:

1. Pre-processing of measured data from the trial projects (see Section 0).
2. Selection of independent variables that have a significant influence on the target variable (heat production or energy demand).
3. Creation of the model and calibration of the hyperparameters of the model using a cross-validation procedure to improve accuracy using the original trial datasets. In machine learning, a hyperparameter is a parameter whose value is used to control the learning process of the model. The hyperparameters that are used for all the models are shown in the Table 4. Cross-validation is a testing procedure that is used to test and validate the effectiveness of a machine learning model. The most common approach, which is also used in this study, consists of splitting the original dataset into five partitions, with each partition carrying 20% of the data. Data in four of the partitions is used to train the model and the data in the remaining partition is used to test it. For each combination of training/testing partitions, the performance of the model is calculated and assessed. In this step, the model’s predictions for heat production and energy demand were compared to the data from Section 0.

**Table 4:** List of the hyperparameters and their values used to run the XGboost models.

base_score = 0.5	n_estimators = 200
booster = ‘gbtree’	n_jobs = 1
colsample_bylevel = 1	nthread = −1
colsample_bynode = 1	objective = ‘reg:squarederror’
colsample_bytree = 0.9	random_state = 0
gamma = 0.3	reg_alpha = 0
importance_type = ‘gain’	reg_lambda = 1
learning_rate = 0.1	scale_pos_weight = 1
max_delta_step = 0	seed = None
max_depth = 4	silent = None
min_child_weight = 4	subsample = 0.9
missing = None
4. Testing the performance of the model.

The variables common to all the models created in this study were:

- The half-hour of the day,
- The day of the week,
- The month of the year,
- The daily average OAT,
- The target variable (the half-hour heat production or energy demand).

**Fig. 5:**
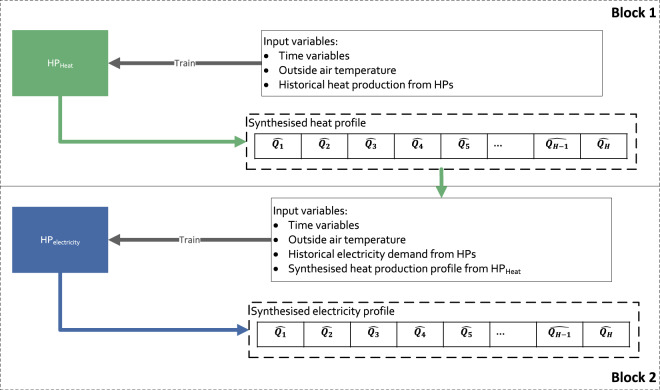
Process to produce half-hourly electricity demand and heat production for HPs with two distinct models *HP*_*heat*_ and *HP*_*electricity*_.

For the case of heat pumps (HPs), for which the COP is impacted by the OAT, the approach shown in Fig. 5 was used to synthesise the energy demand profile. A first model (*HP*_*heat*_) was created to synthesise the heat production profile (block 1) using the list of common variables described previously. The heat production profile produced with *HP*_*heat*_ model was used as an additional variable of a second model (*HP*_*electricity*_) (block 2) to synthesise the electricity demand profile. Besides this additional feature, the *HP*_*electricity*_ model was created following the same process as the *HP*_*heat*_ model.

When producing the data, if the COP is below 1, then the electricity prediction is set to be equal to the heat prediction divided by the average daily COP of the data from Section 0, 2.6 for ASHPS and 2.75 for GSHPS.

**Fig. 6:**
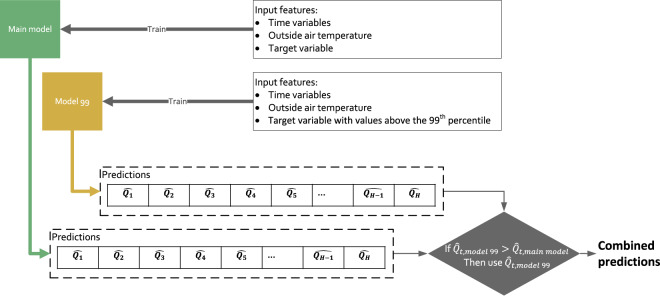
Flow chart of an example model which combines the predictions of two sub-models to improve the accuracy in predicting peaks. ${\widehat{Q}}_{t}$ is the predicted value of a model for time t with t = 1, 2, …, H.

In this study, an ensemble approach was used where the predictions from different sub-models were combined to improve the accuracy of the final predictions. Figure 6 shows an example of this approach with two sub-models: a *Main* model and an additional model, *Model 99*. These sub-models were used to predict the values of a target variable, either heat production or energy demand. The *Main* model was trained on the entire dataset. Meanwhile, *Model 99* was trained on a subset of the dataset that only included the target variable values above the 99^th^ percentile, other values were set to zero. When the predictions from the *Main* model were lower than the predictions from *Model 99*, they were replaced by the predictions from *Model 99*. This was done to improve the predicted peak heat production and peak energy demand. The results are referred to as combined predictions.

When using the *Main* model by itself, the synthesised heat production and energy demand of the heating technologies showed an accurate estimate of the total heat production or energy demand (with less than 1% error) but the peak was underestimated by 6% to 17% (see Table 5). To improve the performance of the method to predict peak heat production and energy demand, five other sub-model combinations were also tested following the approach described in Fig. 6:

1. *Main model* + *Model 99*;
2. *Main model* + *Model 95*;
3. *Main model* + *Model 90*;
4. *Main model* + *Model 95* + *Model 90; and*,
5. *Main model* + *Model 95* + *Model 99*.

**Table 5:** Percentage errors in the total heat produced, or energy consumed (Metric 1) and in the coincidental peak values (Metric 2) of the synthesised data of the models selected for each technology compared to the original data.

Models	Metrics	Main model	Main model+Model 99	Main model+Model 95	Main model+Model 90	Main model+Model 95+Model 90	Main model+Model 95+Model 99
*ASHP*_*heat*_	Metric 1	1%	1%	1%	1%	**1%**	1%
	Metric 2	−11%	−11%	−10%	−9%	−**9%**	−10%
*ASHP*_*electricity*_	Metric 1	1%	1%	1%	1%	**1%**	1%
	Metric 2	−9%	−8%	−6%	−6%	−**5%**	−6%
*GSHP*_*heat*_	Metric 1	−1%	−1%	−1%	−**1%**	−1%	−1%
	Metric 2	−10%	−9%	−8%	−**6%**	−7%	−8%
*GSHP*_*electricity*_	Metric 1	0%	0%	0%	**0%**	0%	0%
	Metric 2	−8%	−7%	−6%	−**5%**	−5%	−6%
*GasBoiler*_*gas*_	Metric 1	0%	0%	**1%**	1%	1%	1%
	Metric 2	−6%	−5%	−**1%**	−2%	0%	−1%
*ResistanceHeater*_*electricity*_	Metric 1	−1%	−1%	0%	**0%**	0%	0%
	Metric 2	−17%	−16%	−13%	−**11%**	−11%	−13%

The models selected to synthesise the dataset produced with this article are highlighted in bold.

*Model 95* refers to a model trained with heat production values above the 95^th^ percentiles, while *Model 90* refers to a model trained with values above the 90^th^ percentiles.

The performance of the models when used to synthesise heat production and the models when used to synthesise energy demand for each technology were analysed using two metrics calculated during the cross-validation procedure:

- The first metric is total heat produced, or energy consumed (the area under the curve).
- The second metric is the coincidental peak for values above the 99% percentile. This error is calculated by comparing the model values in the synthesised dataset with values in the original trial dataset at the same timesteps. The timesteps are drawn from the values of the original dataset above the 99^th^ percentile.

We selected those models with the lowest errors on the two metrics. Table 5 shows the errors on the two metrics for the combinations of models considered. The models selected to synthesise the dataset produced with this article are highlighted in bold red for each technology.

The final synthesised half-hourly heat production and energy demand were normalised. For example, the sum of the values of the synthesised heat production over a year was equal to 1, while the synthesised energy demand was normalised based on the synthesised heat production profiles to maintain the conversion efficiency factor of each technology.

## Data Records

All of the data is available for download on the UK Research Energy Centre platform^6^.

The data is provided in three datasets:

1. ***Annual_heat_demand_LSOA*** dataset, which contains information about dwelling categories and residential heat demand of LSOAs.
2. ***Energy_efficiency_improvements_costs_LA*** dataset, which contains estimated costs to implement energy efficiency measures.
3. ***Half-hourly_profiles_of_heating_technologies*** dataset, which contains half-hourly profiles of average heat production and energy demand of four individual heating technologies.

The ***Annual_heat_demand_LSOA*** dataset includes the following information for all LSOAs in England and Wales:

- The number of units by dwelling categories in 2018. The number of residential electricity meters at LSOA level^12^ was used as a proxy to project the number of dwellings from 2011 (from 2011 Census^13^) to 2018, assuming that the share of each dwelling category had remained unchanged.
- Average annual heat demand before considering energy efficiency measures of each dwelling category calculated using the EPC-based method. To clean the dataset from outliers, only the EPCs of dwellings with a minimum of 15 kWh/m^2^/year for space heating were used as an input to the EPC-based method. This corresponds to the EnerPHit standard, which is the passive house standard for refurbished dwellings. In the final dataset for England and Wales, the outliers above the 99^th^ percentile were replaced by the 99^th^ percentile value.
- Average annual heat demand after considering energy efficiency measures of each dwelling category calculated using the EPC-based method.
- Total residential heat demand before energy efficiency measures based on the number of dwellings in 2018.
- Total annual residential heat demand after energy efficiency measures based on the number of dwellings in 2018.
- Maximum length of heat networks based on the length of the road. The length of the road in each LSOA was calculated from the OS open road data^14^, excluding motorways and roads classified as A roads, which was combined with the boundaries of the LSOAs using GIS software.
- Area of the LSOA.
- Rurality, which is based on urban/rural classification from the census data^13^. There are three possible values: “*Urban”*, “*Village, Town and Fringe”* and *“Hamlet & Isolated Dwellings”*.
- Local authority name based on classification from 2011 and 2019 from the Office for National Statistics (ONS).
- LSOA code based on the 2011 classification^13^.

**Fig. 7:**
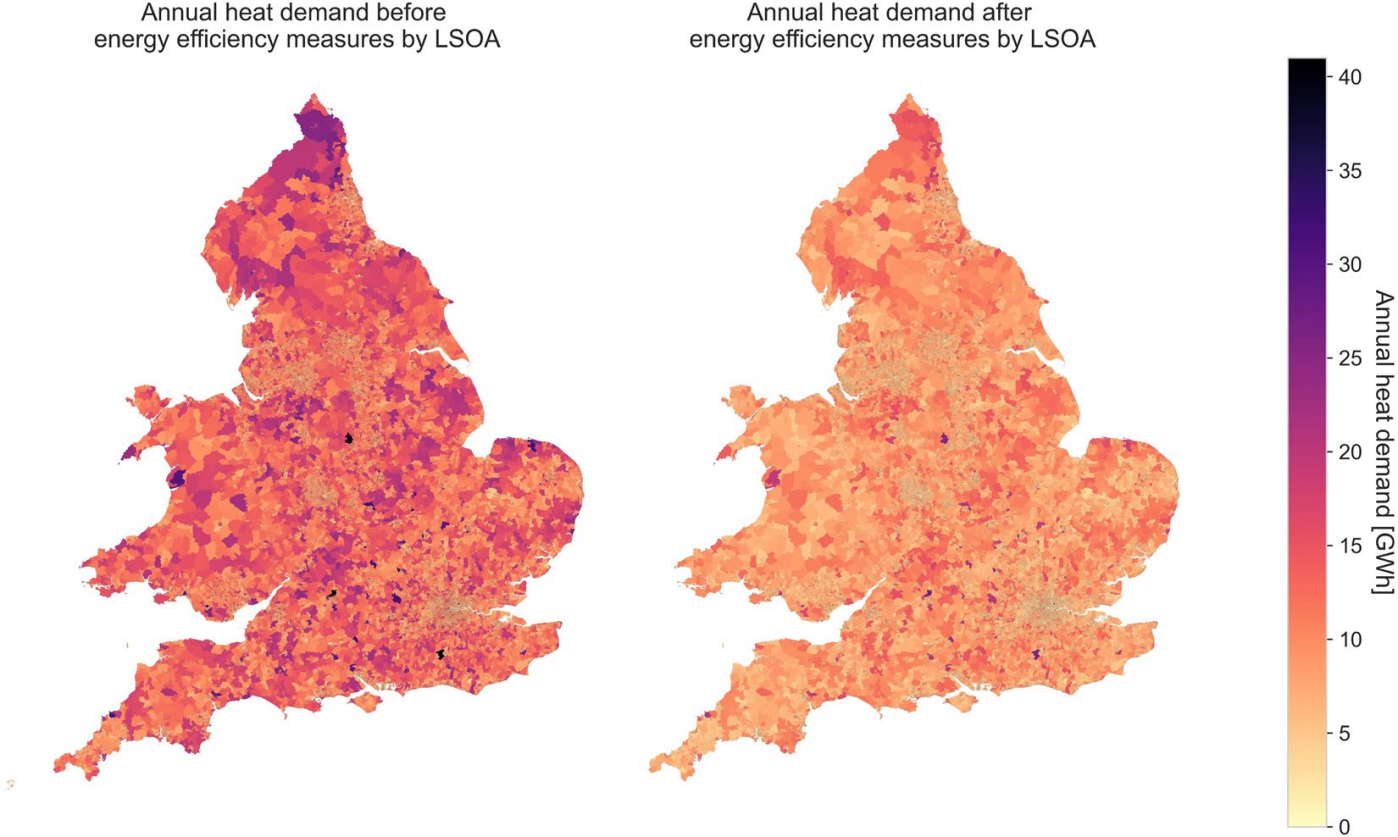
Maps of the annual heat demand in GWh before and after energy efficiency measures at LSOA level of England and Wales based on the number of dwellings in 2018.

Figure 7 shows two maps of England and Wales built using this dataset illustrating the total residential annual heat demand for a typical year at LSOA level before and after energy efficiency measures.

**Fig. 8:**
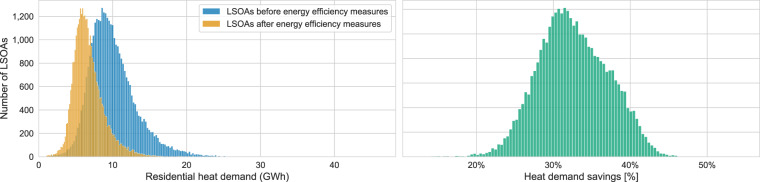
The left-hand chart shows the number of LSOAs based on the total residential heat demand before and after considering energy efficiency measures based on 2018 data. The right-hand chart shows the heat demand savings by implementing the energy efficiency measures.

Figure 8 shows the magnitude of the heat demand savings in more detail by considering energy efficiency measures and the number of LSOAs impacted.

The ***Energy_efficiency_improvements_costs_LA*** dataset includes for each local authority:

- Average cost to improve the energy efficiency of each dwelling category. For each local authority, this was calculated using the recommended energy efficiency measures displayed on EPCs and considering their indicative costs. See Table 6 for further details of the measures included and their costs.

**Table 6:** The costs (£) of energy efficiency measures considered by dwelling types that were used to estimate the costs of energy efficiency improvements in England and Wales.

Costs of energy efficiency measures [£]	Detached	Flat	Semi-detached	Terraced	Source
50 mm internal or external wall insulation	10325	4575	8650	5825	^32^
Add additional 80 mm jacket to hot water cylinder	90	90	90	90	^32^
Cavity wall insulation	815	405	1189	482	^32^
Condensing oil boiler with radiators			4000		^32^
Draughtproof single-glazed windows	200	200	200	200	^33^
Fan assisted storage heaters and dual immersion cylinder	2064	2064	2064	2064	
Fan assisted storage heaters/warm air unit	1564	1564	1564	1564	^34^
Floor insulation	900	0	750	550	^32^
Flue gas heat recovery device in conjunction with boiler	1200	400	800	800	^35^
Heat recovery system for mixer showers	1000	1000	1000	1000	^36^
High heat retention storage heaters	755	580	640	640	^37^
High heat retention storage heaters and dual immersion cylinder	1255	1080	1140	1140	
High performance external doors	2000	2000	2000	2000	^38^
Hot water cylinder thermostat	30	30	30	30	
Increase loft insulation to 270 mm	1627.5	375	1332.5	1067.5	^32^
Biomass boiler	3800	2600	3150	2700	^32^
Condensing gas boiler	3750	1750	2550	2300	^32^
Double-glazed windows	7100	3000	5950	4450	^32^
Secondary glazing to single-glazed windows	4900	1800	3680	3240	^39^
Solar water heating	5000	0	4500	4000	^40^
Time and temperature zone control	150	150	150	150	
Upgrade heating controls	400	400	400	400	^34^
Room-in-roof insulation	2100	2100	2100	2100	^41^
- The number of units by dwelling category in 2018.
- Total cost to implement the energy efficiency measures of all of the dwellings at local authority level based on the average cost of each dwelling category and the number of dwellings in 2018.
- Local authority name based on classification from 2011 and 2019 from the ONS.

**Fig. 9:**
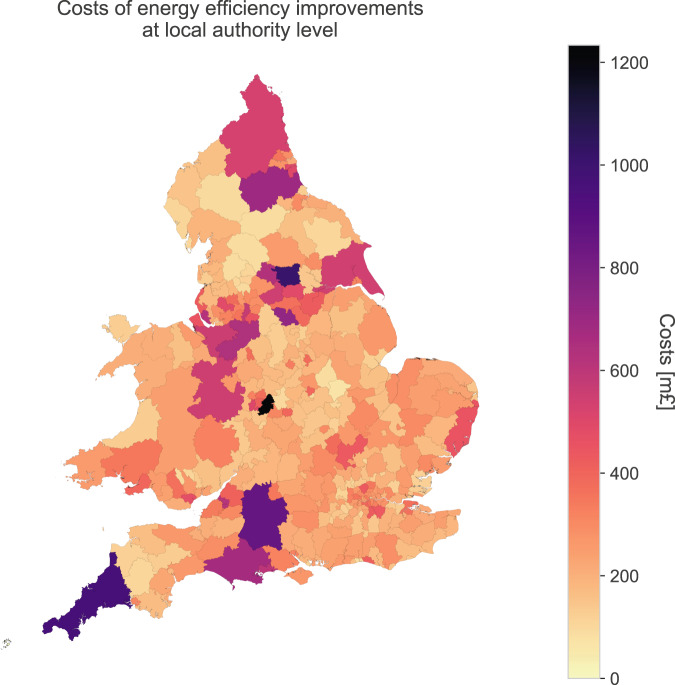
Costs (million pounds) of implementing the energy efficiency measures in dwellings in each local authority in England and Wales.

Figure 9 shows the costs of energy efficiency improvements of the local authorities in England and Wales.

The ***Half-hourly_profiles_of_heating_technologies*** dataset includes:

- The normalised half-hourly heat production and energy demand of ASHPs, GSHPs, resistance heaters and natural gas/hydrogen boilers for a typical UK year. The heat production profiles were normalised such that the sum of the values of the profiles over a year was equal to 1. The energy demand profiles were normalised based on the heat production values to keep the conversion of the efficiency of the technologies. A correction factor was applied to these profiles to account for the difference in OAT between the annual heat demand from the EPC-based method and the daily average OAT profile from 2013. The designed OAT of HPs was assumed to be −3.2 °C. This means that for an OAT of −3.2 °C and above, 100% of the heat demand was supplied by the HPs.
- The daily average UK OAT for the year 2013, which had 1,982 heating degree days, the minimum daily average OAT was −0.8 °C and the maximum 23.2 °C.

**Fig. 10:**
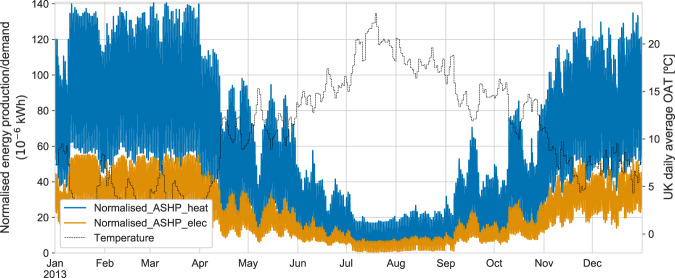
Normalised heat production and electricity demand of an ASHP synthesised using the 2013 daily average OAT profile.

Figure 10 shows the normalised profiles for the ASHP technology. The upper profile (blue) shows the normalised heat production of a pool of ASHPs across a year. The lower profile (orange) is the normalised electricity demand required by these ASHPs to supply the heat shown in the upper profile, which accounts for the COP of the ASHPs that change with OAT.

## Technical Validation

### Comparison with the average residential heat demand by fuels from a study by the centre for sustainable energy

**Fig. 11:**
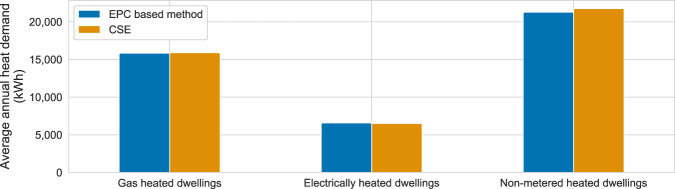
Comparison of the average annual heat demand of dwellings heated with gas, electricity and non-metered fuels from the CSE report^15^ and the EPC-based method aggregated for England and Wales. For non-metered heated dwellings, only detached dwellings were used.

The Centre for Sustainable Energy (CSE) studied the energy usage of different dwellings in Great Britain in 2014 based on 32,700 housing surveys. The outputs of this study included average annual heat demand by heating fuels: gas, electricity and non-metered fuels (e.g., oil and biomass)^15^. Figure 11 shows a comparison of the heat demand from the CSE study with the estimated heat demand by heating fuels produced by EPC-based method in England and Wales.

A comparison of the average annual heat demand shows that the values are in the same range. The largest discrepancy was observed for the non-metered heated dwellings, where the annual heat demand from the CSE study is 2% higher than the output of the EPC-based method applied to non-metered dwellings in England and Wales. There are two main reasons that might explain this difference: first, the accuracy of the heat demand estimated in the EPC of non-metered buildings; and second, the assumptions for the efficiency rating of the heating technologies.

### Comparison with gas demand data

Data on gas consumption in the residential sector at LSOA level in England and Wales is published on the Department for Business, Energy & Industrial Strategy’s (BEIS’s) website^16^. This data was used to estimate heat production by gas boilers, and was compared with heat demand from gas boilers produced by the EPC-based method for all LSOAs in England and Wales.

The BEIS gas consumption data is calculated from gas meter readings and is “weather desensitised” using adjustment factors provided by the gas industry^17^. To align it with the methods used to produce EPC, which is based on models using OAT profiles from the Standard Assessment Procedure (SAP) guidelines^18^, the BEIS gas data at LSOA level was weather corrected using the following steps:

1. Original gas consumption data (before “weather desensitisation”) was calculated using the ratio between the original gas consumption data and the “weather desensitised” gas consumption data for domestic gas demand in England and Wales published by BEIS^19^; and,
2. Weather was corrected using the difference in degree days (using 15.5 °C as a base temperature) between the SAP OAT profile for the UK, which is equivalent to 2,062 degree-days, and the number of degree days in 2018, which is 1,687 and is calculated from the daily average OAT profile from the BMRS report.

The energy consumption statistics published by BEIS^20^ show that 97.5% of residential gas consumption is used for space heating and hot water. A report from Delta EE^10^ suggested an average efficiency of gas boilers of 84%. Hence, for each LSOA, gas consumption data was used to estimate annual heat demand through (Eq. 1):

1 $$Heat\;from\;ga{s}_{LSOA}=BEIS\;gas\;consumptio{n}_{LSOA}\times 97.5{\rm{ \% }}\times 84{\rm{ \% }}$$

**Fig. 12:**
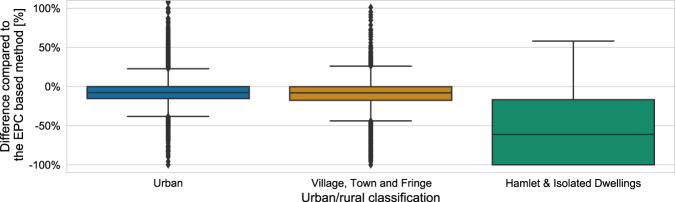
Box plots showing the differences of the heat from gas calculated using BEIS data compared to the heat from gas data from the EPC-based method for the LSOAs in England and Wales based on their rurality classification. The chart was truncated but there are 39 LSOAs with differences above + 100% in “Urban” and “Village, Town and Fringe” areas.

Figure 12 shows the difference between the heat demand in dwellings supplied by gas in 2018 derived from the gas consumption data from BEIS and the EPC-based method of the LSOAs in England and Wales for three levels of rurality. In the LSOAs in “Urban” and “Village, Town and Fringe” areas, the median difference is −8%. This shows a good agreement between the two methods in LSOAs with high density of dwellings. In contrast, the median difference is −61% for LSOAs in the “Hamlet & Isolated Dwellings” area. This large difference is explained by the low density of dwellings connected to gas networks and the low annual heat demand of these LSOAs, which accentuate the differences between the two methods.

Overall, the values of the EPC-based method are mostly greater than the results produced from BEIS data. These differences might be explained by the decrease in residential gas consumption, which has been happening almost continuously since 2010. This is due to a combination of factors, including economic recession, increase in prices, and changes in building stock and household composition, which can be seen in the data from BEIS^12^. This decrease might not be fully reflected in the procedure used to create EPCs and thus may not be captured in the EPC-based method. In addition, BEIS pointed out there was missing or unallocated data, which could result in an underestimation of the gas consumption in LSOAs^21^.

**Fig. 13:**
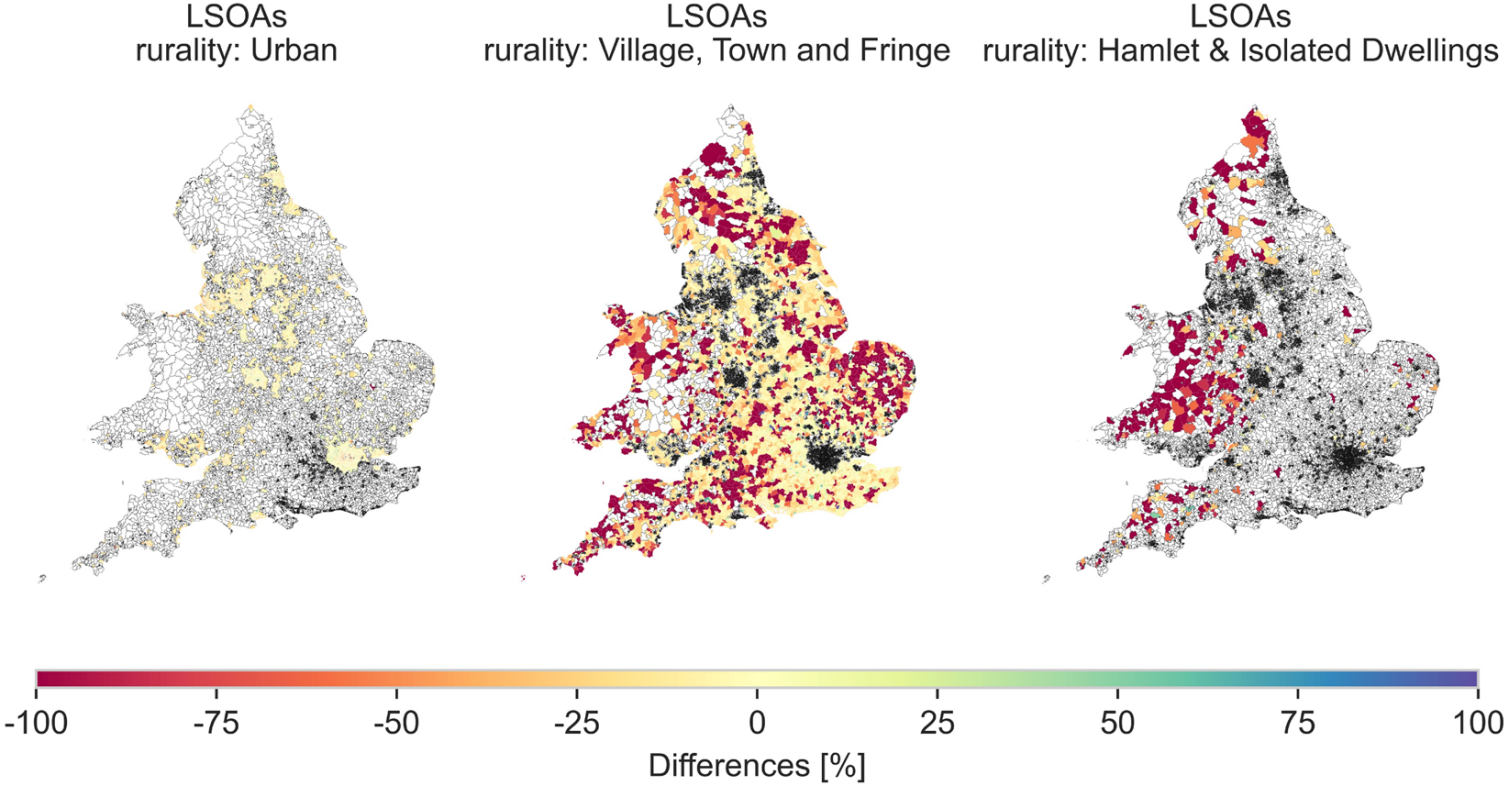
Maps showing differences between the annual heat production by gas boilers calculated from BEIS data and the annual heat production estimated by the EPC-based method, for LSOAs that have different rurality characteristics.

Figure 13 shows that in terms of spatial distribution, the LSOAs in the category of “Hamlet & Isolated Dwellings” are mostly located in Wales, the West and North of England. It represents one percent of the dwellings in England and Wales. The LSOAs in the category of “Village, Town and Fringe” are distributed over all of England and Wales.

Although there are some possible limitations to the EPC-based method in estimating heat from gas in areas with a low density of dwellings, the difference with the BEIS data in more urban areas is on average less than 10%.

### Validation of the estimated coefficient of performance of air-source heat pumps and ground-source heat pumps

The daily average COP of ASHPs and GSHPs were estimated by calculating the ratio of the half-hourly heat production to the electricity demand of ASHPs/GSHPs synthesised in this study and then taking the daily average values. They were validated by comparing them to the values from four sources, as follows:

1. A field test of 23 ASHPs and 49 GSHPs installed in retrofitted dwellings in the UK with data recorded from April 2009 to April 2010. The results^22^ were published by the Energy Saving Trust (EST) for the UK Department of Energy and Climate Change (DECC).
2. The time series of the COP of the ASHPs and GSHPs using radiators as a heat sink for Great Britain, which is published on the Open Power System Data platform^23^.
3. A field test of 21 ASHPs installed in retrofitted dwellings in Germany and recorded for the years 2007 and 2008^24^.
4. A field test of eight ASHPs installed in retrofitted dwellings in Scotland^25^.

For sources 1, 2 and 3, there was no information regarding the relationship between the OAT and the COP of the ASHPs/GSHPs, thus a constant daily average COP was assumed. For source 4, the data was only provided for the range of −5 °C to +15 °C. Only sources 1 and 2 have information for GSHPs.

**Fig. 14:**
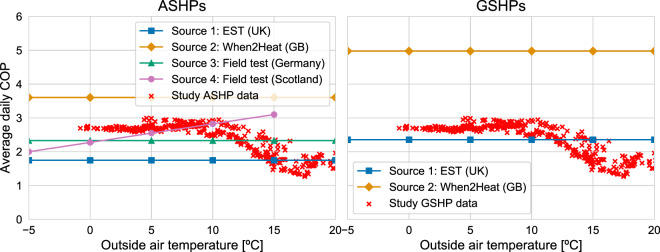
Left-hand panel: the daily average COP of ASHPs from this study and four external sources. Right-hand panel: shows the daily average COP of GSHPs from this study and two external sources.

Figure 14 shows a comparison of the daily average COP of the ASHPs and GSHPs for OAT from −5 °C to +20 °C. The crosses represent the daily average COP of ASHPs and GSHPs of this study. For ASHPs, the maximum is ~2.6 and it reaches ~2.3 at 0 °C. For GSHPs, the maximum is ~3 and it reaches ~2.7 at 0 °C. When the OAT is over +12 °C, the daily average COP starts to decrease. This may be explained by the ASHPs and GSHPs starting to work part-load as heating demand decreases, which decreases their efficiency^26^.

Overall, the daily average COP values of this study are in the same range as the values that have been published from the external sources for ASHPs and GSHPs, although the GSHP’s COP value from Source 2 is much higher than the values from this study. The authors of the Source 2 report that they used a correction factor to increase the validity of their datasets based on a comparison with the COP of HPs from a field test in Germany.

Some of these differences may be explained by the impact of the choice of the heat sink (e.g., underfloor heating or radiators) on the efficiency of the system^23^, the OAT when the data was recorded or the OAT profile used for the modelling. For an OAT above + 15 °C, the decrease in efficiency due to the HPs working part-load was not captured by the other studies.

## Usage Notes

The following subsections discuss the applicability and the limitations of the dataset.

### Applicability

The three datasets of this study were intended to allow researchers and other stakeholders interested in heat decarbonisation in England and Wales to:

- Develop heat decarbonisation pathways for local areas.
- Produce an estimate of the annual heat energy production and peak energy demand at half-hourly/daily/monthly resolution.
- Study the viability of district heating schemes.

**Fig. 15:**
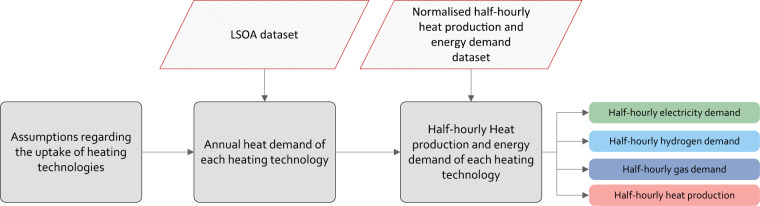
Diagram showing how the datasets can be used to produce half-hourly heat production and energy demand profiles.

Figure 15 shows an example of how the database was used to synthesise profiles for a decarbonisation pathway in an area. This entailed three main steps:

1. Describing assumptions regarding the uptake of heating technologies. For instance, will all the dwellings using gas boilers in 2018 use hydrogen boilers? or, will they use GSHPs, ASHPs?
2. Calculating the annual heat needed to be produced by each heating technology.
3. Distributing the annual heat demand over the year using the normalised half-hourly heat production and energy demand of each technology.

### Limitations

The limitations of the database will be discussed in this section.

#### Annual heat demand

The assumptions that were used to project the number of dwellings from 2011 to 2018 may not accurately capture the share of each dwelling category in 2018. On average, each LSOA has seen its number of dwellings increase by 9% between 2011 and 2018. However, no information was found to estimate the increase in each dwelling category.

There are known limitations to EPCs because of the methods used to produce them and the quality of the recorded data^27^. Furthermore, EPCs assume a standard occupancy of the dwellings, and thus do not consider potential differences in the behaviour of people and other socio-economic factors. There are also limitations due to the number of EPCs available. In this study, around 6 million EPCs were used, whereas there were ~25 million dwellings in England and Wales in 2018. These factors can impact the accuracy of the estimated annual heat demand of the dwellings shown in the database.

The estimated annual heat demand is an average of the values from all of the EPCs belonging to the same dwelling category. Consequently, there could be a large variation between different individual dwellings in the same dwelling category within an LSOA, which is not captured in the database.

#### Half-hourly heat production and energy demand profiles

The data from the trial projects that were used to train the machine learning models for each technology may not provide a good representation of how the heating technologies are controlled or of the behaviour of the occupants regarding heating. This is particularly true for the ASHPs and GSHPs datasets, which are based on relatively small samples of only social housing dwellings (<700 in total).

A better parametrisation of the XGboost algorithm or other type of model (e.g., artificial neural networks, support vector machines, etc.) may provide better accuracy when representing the peaks in the profiles compared to the current models.

#### Peak energy demand

There are three main uncertainties in using the data produced in this study to estimate the peak energy demand of a system: the timing, the magnitude, and the impact of the diversity factor.

The peak energy demand of a system needs to be calculated using a robust methodology that may consider parameters such as extreme weather events, time of the year (e.g., holidays, day of the week, etc.) and potential behavioural change (e.g., people staying at home because of lockdowns).

The timing of the peak energy demand may not be accurately reflected in the profiles of this study. These profiles were created based on the daily average OAT profile of the year 2013, which only includes a few days with daily average OATs below 0 °C and the minimum daily average OAT observed was −0.8 °C. Hence, it does not provide information regarding what the shape of the profiles would be for a more extreme event, such as 1-in-20 peak day.

The diversity factor represents the number of units of a system that are running at the same time. It is defined as shown in Eq. 2:

2 $$D=\frac{Actual\;energy\;demand}{Maximum\;energy\;demand\;if\;all\;the\;units\;are\;running\;at\;the\;same\;time}$$

The amount of diversity at each time step was embedded in the profiles of this study because the original training datasets that were used to train the models were calculated based on the average demand/production of many units. However, if there is a need to calculate the peak energy demand of a LSOA where there are few units installed of a kind of technology (e.g., ASHPs, GSHPs, etc.), then there may be limited diversity and thus the profiles that are used may not adequately represent the shape of the demand. In these cases, it may be better to adopt a more conservative approach and consider no diversity, and instead calculate the peak demand as the number of units in the area multiplied by their rated capacity.

The link between the number of units installed and the amount of diversity to be considered will vary between the heating technologies and the type of user. For example, Love *et al*. used the heat pump datasets from this study and showed that the diversity would not change significantly when more than ~200 units are considered together^28^.

## Data Availability

The Python code that we used to produce the datasets presented in this paper is published at https://github.com/AlexandreLab/UKERC-data.
